# Functional Reorganization After Four-Week Brain–Computer Interface-Controlled Supernumerary Robotic Finger Training: A Pilot Study of Longitudinal Resting-State fMRI

**DOI:** 10.3389/fnins.2021.766648

**Published:** 2022-02-11

**Authors:** Yuan Liu, Shuaifei Huang, Zhuang Wang, Fengrui Ji, Dong Ming

**Affiliations:** Academy of Medical Engineering and Translational Medicine (AMT), Tianjin University, Tianjin, China

**Keywords:** supernumerary robotic finger, resting-state fMRI, fALFF, ReHo, DC, neuroplasticity

## Abstract

Humans have long been fascinated by the opportunities afforded through motor augmentation provided by the supernumerary robotic fingers (SRFs) and limbs (SRLs). However, the neuroplasticity mechanism induced by the motor augmentation equipment still needs further investigation. This study focused on the resting-state brain functional reorganization during longitudinal brain–computer interface (BCI)-controlled SRF training in using the fractional amplitude of low-frequency fluctuation (fALFF), regional homogeneity (ReHo), and degree centrality (DC) metrics. Ten right-handed subjects were enrolled for 4 weeks of BCI-controlled SRF training. The behavioral data and the neurological changes were recorded at baseline, training for 2 weeks, training for 4 weeks immediately after, and 2 weeks after the end of training. One-way repeated-measure ANOVA was used to investigate long-term motor improvement [*F*(2.805,25.24) = 43.94, *p* < 0.0001] and neurological changes. The fALFF values were significantly modulated in Cerebelum_6_R and correlated with motor function improvement (*r* = 0.6887, *p* < 0.0402) from t0 to t2. Besides, Cerebelum_9_R and Vermis_3 were also significantly modulated and showed different trends in longitudinal SRF training in using ReHo metric. At the same time, ReHo values that changed from t0 to t1 in Vermis_3 was significantly correlated with motor function improvement (*r* = 0.7038, *p* < 0.0344). We conclude that the compensation and suppression mechanism of the cerebellum existed during BCI-controlled SRF training, and this current result provided evidence to the neuroplasticity mechanism brought by the BCI-controlled motor-augmentation devices.

## Introduction

The hands and fingers are the important mediums for humans to interact with the outside world and have a well-established functional representation in the brain (Jones and Lederman, 2006; Dall’Orso et al., 2018; Arcaro et al., 2019). Scientists are currently focusing on the hand or arm motor augmentation device like the supernumerary robotic fingers (SRF) and even the entire limbs (SRL) (Llorens-Bonilla et al., 2012; Parietti and Asada, 2013; Parietti et al., 2014; Prattichizzo et al., 2014; Wu and Asada, 2015; Hussain et al., 2017a,b,c; Kieliba et al., 2021). These devices have changed the way our inherent limbs interact with the external environment and bring some effects on the brain and the corticospinal motor synergies (Kieliba et al., 2021; Rossi et al., 2021). However, despite motor augmentation caused by these devices can be clearly observed in behavioral experiments, little notice is given to the brain neuroplasticity mechanism. Here, we used the brain–computer interface (BCI)-controlled SRF system to investigate longitudinal neuroplasticity changes in 4 weeks by using resting-state fMRI (rs-fMRI) local metrics like ALFF, ReHo, and DC.

In the present research on the SRF, the motor augmentation effects were clearly investigated in behavior measurement. For normal people, the extra robotic finger can enhance manipulation dexterity and enlarge the workspace of humans, like grasping a larger-sized object using one hand or completing two-handed collaboration tasks using one hand (Prattichizzo et al., 2014; Wu and Asada, 2015; Kieliba et al., 2021). For patients, the extra robotic finger can compensate missing grasping abilities and help rehabilitation training, like assistance in grasping the cup or manipulation dexterity training for the paretic hand (Hussain et al., 2017a,b,c). However, there was only some preliminary research focus on the brain neuroplasticity effect caused by SRF. From task-based fMRI analysis, it was clearly found that the bilateral cingulate cortex, bilateral superior parietal lobule, left inferior parietal lobule, and right middle frontal gyrus have greater neural representation in the finger opposition task after 2 days of SRF training (Hussain et al., 2017b). The biological hand neural representation in the sensorimotor cortex will generate a shrinkage after 5 days of third thumb wearing training (Kieliba et al., 2021). In addition, healthy humans wearing the SRF will rapidly reshape the pattern of corticospinal outputs toward the forearm and hand muscles governing imagined grasping actions of different objects after a few minutes of training (Rossi et al., 2021) and suggesting that human beings are open to very quick welcoming emerging augmentative bioartificial corticospinal grasping strategies. However, these studies did not pay attention to the long duration training effect on the resting state neuroplasticity of the brain, and these results are affected by the control method of the SRF.

Different from the EMG control (Kieliba et al., 2021) or toe switch control (Hussain et al., 2017b) that required residual motor function, the brain–computer interface (BCI) has been developed to transmit autonomous control intentions to corresponding external execution devices such as robots, orthosis, and functional electrical stimulation (Yuan et al., 2020). A previous study has proven that the human brain has the ability to bear the load of the supernumerary finger from the research of polydactyly subjects (Mehring et al., 2019) and the six-finger illusory perception creation (Newport et al., 2016; Cadete and Longo, 2020). The motor imagery (MI) technology based on BCI has great advantages of transmitting human intentions into the control of the external devices proven in the research of the third arm (Penaloza and Nishio, 2018). From task-based fMRI analysis, MI consistently recruits the frontoparietal network and the subcortical and cerebellar regions (Hetu et al., 2013). As for the reason that MI possesses a similar activation of the motor area during the motor execution, it has been widely used in clinical rehabilitation (Eaves et al., 2014). Clinical studies have found that functional connectivity between sensorimotor regions was significantly modulated after motor imagery training of the own inherent inborn limbs of stroke patients (Zhang et al., 2016). In addition, the rehabilitation neuroplasticity effect has also been fully proved in combination with MI and rehabilitation equipment (Kim et al., 2016; Biasiucci et al., 2018; Wang et al., 2018; Yuan et al., 2021). A study has found that the MI-guided robot-hand training robot has significantly modulated the time variability of the sensory–motor areas, attention network, auditory network, and default mode network in stroke patients than the no MI-guided training group (Wang et al., 2018), and training promotes the recruitment of selected brain areas and facilitates neuroplasticity by providing feedback on the intended movement (Cervera et al., 2018). However, there is currently no neuroplasticity research on the MI-controlled supernumerary robotic limb training. Here, we used the BCI-controlled SRF system based on MI mechanism to investigate the resting state changes of the human brain.

Resting-state functional magnetic resonance imaging (rs-fMRI) is a promising tool to investigate functional alterations in the human brain, which takes into account the advantages of both spatial resolution and time resolution, and also has unique advantages in clinical conditions because it does not require participants to engage in cognitive activities (Biswal et al., 1995; Fox and Raichle, 2007). Although the majority of analytic techniques [functional connectivity (FC) (Friston, 1994), graph theory, independent component analysis (ICA), etc.] for rs-fMRI data characterize the function of the brain network, the local dynamics cannot be fully addressed with these approaches (Lv et al., 2019). Several methods have been proposed to characterize the local dynamic properties of the rs-fMRI signal: fractional amplitude of low-frequency fluctuation (fALFF) (Zou et al., 2008), regional homogeneity (ReHo) (Zang et al., 2004), and degree centrality (DC) (Buckner et al., 2009). The fALFF measures the relative predominance of low-frequency amplitude to the amplitude of all oscillations across the entire power spectrum (Zou et al., 2008). ReHo was proposed to measure the synchronization of the voxel time courses with the neighboring voxels based on the hypothesis that voxels within a functional brain area synchronize their metabolic activity depending on specific conditions (Zang et al., 2004). DC mapped the degree of intrinsic FC across the brain in order to reflect a stable property of cortical network architecture at the voxel level (Buckner et al., 2009). These three voxel-wise metrics define brain functional characteristics from different perspectives (single voxel, neighboring voxels, and whole brain) and present the progressive relationship (Lv et al., 2019).

This current study aims to fill this gap by investigating the functional reorganization of 4 weeks of BCI-controlled SRF training based on the new supernumerary robotic finger imagery paradigm. Specifically, we sought to determine how SRF training influence the local function by using three local metrics (fALFF, ReHo, and DC) and whether those local changes (if observed) are associated with behavioral performance of the participants. These findings in this study may bring some insights into the mechanism of neuroplasticity brought by the BCI-controlled augmentative device.

## Materials and Methods

### Participants

Ten participants (4 females; aged 21.5 years; range 20–23 years) were recruited from Tianjin University. Participants were all right handed (laterality quotient 0.89 ± 0.09; range 0.60–1.0) as assessed by the Edinburgh Handedness questionnaire. Participants gave informed consent, and the study was approved by the Tianjin University Human Research Ethics Committee.

### Training System and Intervention Protocols

**FIGURE 1:**
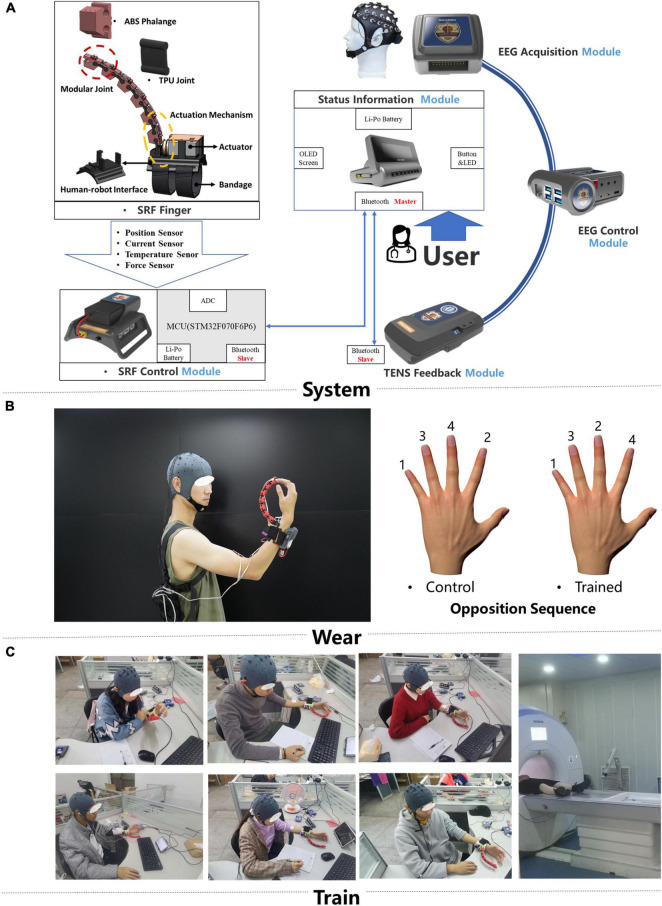
Training system and intervention protocols. **(A)** The hardware design of the brain computer interface (BCI)-controlled supernumerary robotic finger (SRF) system and the signal transmission diagram **(B)**. The display of the portable SRF and behavioral measure paradigm. **(C)** Scene of subjects wearing SRF training.

A self-designed brain control supernumerary robotic finger (SRF) system was used in this training (Figure 1A; Liu et al., 2021a,b). The whole system contained six modules: EEG acquisition, EEG control, SRF control, SRF finger, TENS feedback, and status information module. The EEG acquisition module uses the module OPENBCI to collect the eight channels of EEG signals of FC1, FC2, FCZ, etc., with a sampling frequency of 250 Hz. Then the EEG signal is resampled, filtered (8- to 13-Hz bandpass, 50-Hz notch), re-referenced, ICA, and time-frequency features extracted. Finally, the preprocessed EEG signal is imported into the convolutional neural network (CNN) to obtain the training model. The CNN was formed by two convolutional layers, a pooling layer and two fully connected layers. The first layer of convolution kernel was to extract the time characteristics of each channel and frequency band of EEG signal. The second layer of convolution kernel was to integrate the eight channel features and extract the signal spatial features. Finally, the two fully connected layers were used to realize the training of the two-feature classification model of MI state and resting state (Dose et al., 2018). A novel “sixth-finger” motor imaginary (MI) paradigm is performed to provide the SRF natural control. Participants wear the SRF and imagined the SRF finger opposing with the inborn inherent finger from the first-person perspective. Based on the developed “sixth-finger” MI decoding algorithm, the difference between the MI and resting (rest) state of the EEG signal can be classified, and the training model of the MI state is used for online classification. The EEG characteristic investigation interrelated with the new MI paradigm is investigated in detail in another paper of our team (Liu et al., 2021b).

The SRF finger module is driven by a single actuator and has one DOF (degree of freedom) to perform the flexion and extension. When the system captures the MI signal, the SRF finger module will flex, and participants will move their inborn inherent finger to cooperate with the SRF finger. In the SRF fingertip, a capacitive sensor detects the finger force signal and give the feedback to the system to make the SRF finger extend. At the same time, the TENS feedback module will release a 0.2-s electrical stimulation (duty factor: 50%, frequency: 5 Hz, voltage: 10 V) to the median nerve of the forearm wearing the SRF.

The SRF finger was worn on the left hand, and the reason for using subdominant hands (left hand) instead of dominant (right hand) is the better anti-interference ability for daily life activities (Figure 1C). During the training, participants were asked to sit in front of a table to keep their bodies relaxed. All participants received a 20-session BCI-controlled SRF training in 4 weeks with an intensity of five sessions per week and 1 h per session. During each training session, the subject was required to imagine the MI paradigm. When the system completes one MI trigger, the SRF finger will bend four times and cooperate with the inherent four fingers to complete a round of SRF-finger opposition task. The training opposition sequence is little, middle, ring, index (Sale et al., 2017; Figure 1B), and the intermittent breaks every 10 repetitions were given to avoid fatigue.

### Data Acquisition

Behavioral and rs-fMRI measures were obtained in four time periods: before training (t0), training for 2 weeks (t1), immediately after 4 weeks of training (t2), and 2 weeks after the intervention (t3).

#### Behavioral Measure

The performance of participants on the SRF-finger opposition tasks was evaluated by the number of correct sequences completed in 30 s. The performance was documented online with a handheld video camera and quantified offline. Participants performed both trained and control sequences (Sale et al., 2017) using their left hand with SRF, as shown in the opposition sequence figure of Figure 1B. The trained sequence (order: little, middle, ring, index) was used for every day training and collected in data acquisition periods. The control sequence (order: little, index, ring, middle) was only acquired in data acquisition periods to investigate whether training induced any spill-over of effects to a novel sequence. This method has been used in other finger training tasks (Sale et al., 2017). Furthermore, prior to the quantification of baseline performance of the sequences (before training), participants were given a brief period of time (two to three sequences) to practice the two sequences.

#### Image Data Acquisition

All participants were scanned with a 3T Siemens MAGNETOM Skyra scanner at Tianjin Huanhu Hospital (Department of Neurosurgery, Tianjin, China). Resting-state fMRI images were acquired using T2-weighted gradient-echo planner imaging (EPI) sequence (TR = 2,000 ms, TE = 30 ms, flip angle = 90°, FOV = 220 × 220 mm^2^, matrix = 64 × 64, slice thickness = 3 mm, gap = 0 mm, voxel size = 3.5 × 3.5 × 4 mm^3^, acquisition time = 8:06 min). During the scanning, participants were instructed to stay still and keep their eyes closed without falling asleep. In addition, a T1-weighted structural image was acquired for each participant using the MPRAGE sequence (TR = 2,000 ms, TE = 2.98 ms, flip angle = 9°, FOV = 256 × 256 mm^2^, matrix = 256 × 256, slice thickness = 1 mm, voxel size 1 × 1 × 1 mm^3^, acquisition time = 4:26 min).

### Image Processing

#### Resting-State fMRI Data Preprocessing

Resting-state fMRI data were processed using SPM12 and RestPlus (Jia et al., 2019) including (1) removing the first 10 time points to make the longitudinal magnetization reach steady state and to let the participant get used to the scanning environment, (2) slice timing to correct the differences in image acquisition time between slices, (3) head motion correction, (4) spatial normalization to the Montreal Neurological Institute (MNI) space via the deformation fields derived from tissue segmentation of structural images (resampling voxel size = 3 mm × 3 mm × 3 mm), (5) spatial smoothing with an isotropic Gaussian kernel with a full width at half maximum (FWHM) of 6 mm, (6) removing linear trend of the time course, (7) regressing out the head motion effect (using Friston 24 parameter) from the fMRI data (Friston et al., 1996), and (8) band-pass filtering (0.01–0.08 Hz). One participant was excluded from further analysis due to large head motion (more than 3.0 mm of maximal translation in any direction of x, y, or z or 3.0° of maximal rotation throughout the course of scanning).

#### Fractional Amplitude of Low-Frequency Fluctuation Calculation

After data preprocessing (exclude preprocessing part 8: band-pass filtering), the time series for each voxel was transformed into the frequency domain using a fast Fourier transform, and the power spectrum was then obtained. The averaged square root was obtained across 0.01–0.08 Hz at each voxel, and this value was regarded as the power and then a ratio of the power of each frequency at the low-frequency range (0.01–0.08 Hz) to that of the entire frequency range (0–0.25 Hz) as the fALFF value (Zou et al., 2008).

#### Regional Homogeneity Calculation

After preprocessing (exclude preprocessing part 5: spatial smoothing), ReHo maps were produced by the Kendall’s coefficient of concordance (KCC) of the given voxel time series with its nearest 26 neighbors. The formula is as follows:


(1) $$W=\frac{\sum {\left(R_{i}\right)}^{2}-n{\left(\bar{R}\right)}^{2}}{\frac{1}{12}K^{2}\left(n^{3}-n\right)}$$

where W is the KCC among the given voxels, ranging from 0 to 1; *R_i_* is the sum rank of the *i*th time point; $\bar{R}$ = [(n+1)K]/2 is the mean of *R*_*i*_′s; K is the number of the time series within a measured cluster (*K* = 7, 19, and 27, respectively. 27 in the current study); and n is the number of ranks. This method measures the local synchronization of the given time series (Zang et al., 2004).

#### Degree Centrality Calculation

Degree centrality is defined as the sum of weights from edges connecting to a node. After preprocessing (exclude preprocessing part 5: spatial smoothing), Pearson’s correlation of time series was performed between each voxel, and the correlation coefficients were summed up for each voxel after taking the threshold (*r* ≥ 0.25), and then a weighted DC was obtained for each voxel. The weighted DC of each voxel was further divided by the global mean weighted DC of each individual for group comparison (Zuo et al., 2012). This method is also called the function connectivity strength (FCS).

### Statistical Analysis

The performance of SRF-finger opposition sequences was analyzed using one-way repeated measures ANOVA at time level (t0, t1, t2, and t3) and *post-hoc* analyses were performed using Bonferroni’s *post-hoc* test. At the same time, the correct number changes of the SRF-finger opposition sequences were counted and used to correlate with the MRI data. Statistical analyses were performed using SPSS 22 (IBM SPSS Statistics, NY, United States) with the significance level set at *p* < 0.05.

For longitudinal comparisons, one-way repeated-measure ANOVA and Bonferroni’s *post-hoc* test was used to explore the significance of differences in ALFF, ReHo, and DC changes among various time-related subgroups. In addition, multiple comparisons were corrected using the GRF correction (the voxel-wise, *p* < 0.005; the cluster-wise, *p* < 0.05) in the RestPlus toolbox. For any measure (fALFF, ReHo, and DC) showing training-related alterations, a Pearson correlation analysis was used to assess its associations with behavioral performance of the participant. The correlations were considered significant at a threshold of *p* < 0.05.

## Results

### Behavioral Performance

**FIGURE 2:**
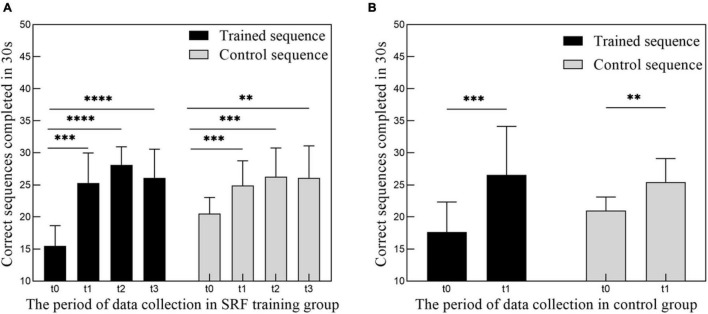
**(A)** Increase in performance of motor training tasks following 4 weeks of SRF training. Group data (*n* = 10) showing number of correct sequences (trained and control sequence) performed in 4 periods (t0, t1, t2, t3) of data collection. **(B)** Increase in performance of motor training tasks following 2 weeks of finger movement training. Group data (*n* = 9) showing number of correct sequences (trained and control sequence) performed in 2 periods (t0, t1) of data collection. Data represent mean ± SEM, * indicates a significant difference in the two groups. ^**^p < 0.01, ^***^p < 0.005, ^****^p < 0.001.

Following training, there was a significant improvement in the number of sequences completed in the 30-s period [Figure 2A; *F*(2.805,25.24) = 43.94, *p* < 0.0001]. The number of correct sequences completed on the trained sequence significantly increased from t0 to t1 (effect of time *p* < 0.0001), reached to the maximum value at the period of t2, and decreased slightly at the period of t3. At the same time, the control sequence showed the same trend with the trained sequence [*F*(2.267,20.40) = 14.01, *p* < 0.0001].

### Longitudinal Analysis Contains Fractional Amplitude of Low-Frequency Fluctuation, Regional Homogeneity, and Degree Centrality

**FIGURE 3:**
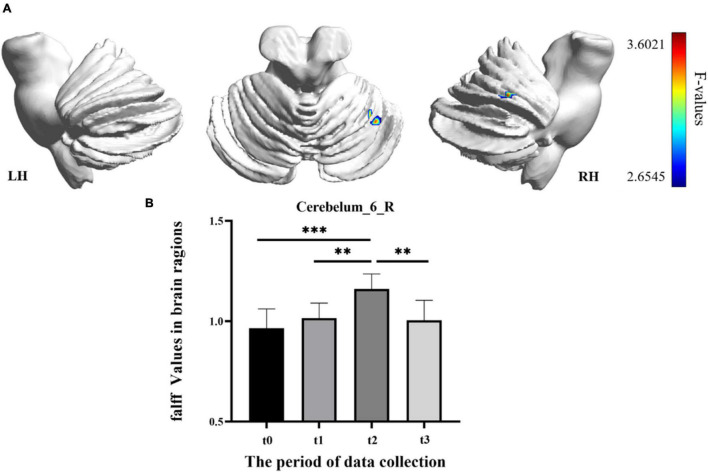
The fractional amplitude of low-frequency fluctuation (fALFF) differences among different time-subgroups. **(A)** One-way repeated-measure ANOVA showed brain regions with fALFF differences among four subgroups in the Cerebelum_6_R. The color bar indicated the F scores. **(B)** Bar plots showed the fALFF values of the Cerebelum_6_R at four different time-subgroups. Cerebelum_6_R, right cerebellum posterior lobe VI; t0, t1, t2, and t3, different time subgroups; LH, left hemisphere; RH, right hemisphere, * indicates a significant difference in the two groups. *p < <0.05, ^**^p < 0.01, ^***^p < 0.005.

In the result of fALFF, participants exhibited a significantly altered right cerebellum posterior lobe (Cerebelum_6_R) after the repeated-measure ANOVA test (GRF correction, voxel *p* < 0.005, cluster *p* < 0.05, cluster size > 31 voxels) (Figure 3 and Table 1). In addition, the fALFF values in Cerebelum_6_R significantly increased during the training period (t0 to t2), reached to the maximum at t2 period, and decreased significantly in the follow-up period (t3 to t4) (*post-hoc* Bonferroni test, all *p* < 0.01).

**TABLE 1:** Brain regions with longitudinal fALFF and ReHo changes.

			MNI coordinate (mm)		
Regions	Cluster size	Peak *T* value	X	Y	Z
**fALFF**					
Cerebelum_6_R	14	3.6021	36	−54	−24
**REHO**					
Cerebelum_9_R	39	4.1268	15	−54	−30
Vermis_3	81	4.0347	0	−30	−6

**FIGURE 4:**
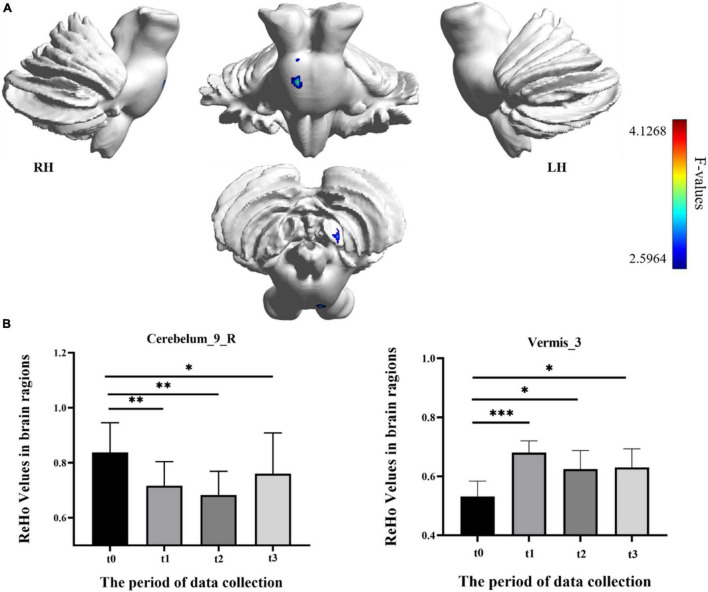
Regional homogeneity (ReHo) differences among different time-subgroups. **(A)** One-way repeated-measure ANOVA showed brain regions with ReHo differences among four subgroups in the Cerebelum_9_R and Vermis_3. The color bar indicated the F scores. **(B)** Bar plots showed the ReHo values of the Cerebelum_9_R at four different time-subgroups. Cerebelum_9_R, right cerebellum posterior lobe IX; Vermis_3, cerebellum anterior lobe III; t0, t1, t2, and t3, different time subgroups; LH, left hemisphere; RH, right hemisphere, * indicates a significant difference in the two groups. *p < <0.05, ^**^p < 0.01, ^***^p < 0.005.

In the result of ReHo, participants exhibited a significantly altered right cerebellum posterior lobe IX (Cerebelum_9_R) (GRF correction, voxel *p* < 0.005, cluster *p* < 0.05, cluster size > 39 voxels) and right cerebellum anterior lobe III (Vermis_3) (GRF correction, voxel *p* < 0.005, cluster *p* < 0.05, cluster size > 81 voxels) after the repeated-measure ANOVA test (Figure 4 and Table 1). In Cerebelum_9_R, the ReHo values showed a decreased trend during training (t0 to t2) and reached the minimum at t2 period. Then it increased (not significantly) in the follow-up period (t2 to t3) but still significantly lower than the baseline stage (t0) (*post-hoc* Bonferroni test, all *p* < 0.05). In Vermis_3, the ReHo values significantly increased to the maximum after 2 weeks of training (t0 to t1) and decreased in the next 2 training weeks thought not significantly. In the period of t3, the ReHo values in Vermis_3 kept steady with t2 but were significantly higher than the baseline stage (t0) (*post-hoc* Bonferroni test, all *p* < 0.05).

No regions showed significant differences in DC after the repeated-measure ANOVA test between different time groups (GRF correction, voxel *p* < 0.005, cluster *p* < 0.05, cluster size > 28 voxels).

### Correlation With Behavioral Function

**FIGURE 5:**
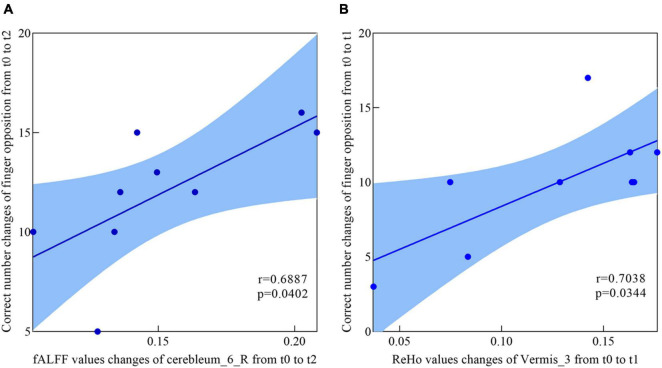
Correlations between fALFF values, ReHo values, and behavioral scores. **(A)** The participants showed a significantly positive correlation between the fALFF values increased in Cerebelum_6_R and the enhancement of behavioral performance (the changes of in correct finger opposition number) from t0 to t2. **(B)** Significantly positive correlation between the ReHo values increased in Vermis_3 and the enhancement of behavioral performance (the changes of in correct finger opposition number) from t0 to t1.

The correlations between the metric changes and behavioral improvement are shown in Figure 5. We mainly compared the behavioral correlations corresponding to the time periods when the rs-fMRI results changed most significantly. We found a significantly positive correlation between fALFF values change in the Cerebelum_6_R and improvement in the behavioral performance (the change in correct finger opposition number) from t0 to t2 (*r* = 0.6887, *p* < 0.0402). The ReHo values of the Vermis_3 also showed a significantly positive correlation with the improvement in the behavioral performance from t0 to t1 (*r* = 0.7038, *p* < 0.0344).

## Discussion

In this study, we investigated the long-term functional reorganization from the rs-fMRI perspective after BCI-controlled supernumerary robotic finger training. The brain regional changes could be identified sensitively from progressive perspectives using these three rs-fMRI analysis metrics: fALFF, ReHo, and DC. Significant changes were observed using fALFF and ReHo analysis, and no changes were found in the DC analysis. The fALFF analysis showed that Cerebelum_6_R was comparatively more active after training, and the fALFF values of Cerebelum_6_R were significantly correlated with motor function improvement. The ReHo analysis showed that Cerebelum_9_R and Vermis_3 were changed comparatively during training, and the ReHo values in Vermis_3 significantly correlated with motor function improvement. To the best of our knowledge, this is the first study to explore BCI-SRF training-induced neural modulation effect from resting state in a longitudinal manner.

Robot-assisted equipment has shown the potential to augment and restore the motor function after training whether in normal people or patients (Yuan et al., 2020; Kieliba et al., 2021). Our results also validated the long-term effect of BCI-controlled robot finger training on normal subjects. It can be clearly found that after 4 weeks of training, not only the operation ability of the training sequence was significantly increased but also the learning ability of the control sequence was significantly increased (as shown in Figure 2). Significant improvement in the control sequence may be related with the generalization of learning, which was correlated with the interaction with the primary visual or motor cortices encoding the stimuli or movement memories (Censor, 2013). However, the present study found no significant changes in the motor and visual cortex, which may be related with the closed loop control system, which has sensory feedback, which will reduce the dependence of the brain on visual resources (Kato et al., 2009). It may also be related with the simplicity of the training task. Future research should be detailed in this generalization phenomenon. This significant improvement of the behavioral results also indicated the effectiveness and acceptance of people to the whole SRF system, which integrated the neuroplasticity of the MI (Varkuti et al., 2013; Jeunet et al., 2019) and the effect of the close loop sensory feedback (Kato et al., 2009; van Dokkum et al., 2015).

Resting-state fMRI is widely used in clinical research because of its unique advantages, which require less participants to engage in cognitive activities (Fox and Raichle, 2007). The fALFF, ReHo, and DC, three voxel-wised metrics, define brain functional characteristics from different perspectives and present the progressive relationship (Zang et al., 2004; Zou et al., 2008; Buckner et al., 2009). For a single voxel, fALFF characterizes neural activity intensity of the single voxel, ReHo reveals the importance of this voxel among the nearest voxels, while DC portrays the importance of this voxel in the whole brain (Lv et al., 2019). In this study, fALFF and ReHo had significant results, which indicated that SRF training mainly modulated brain function from single-voxel level and local level. However, the DC metric had no significant results in the longitudinal ANOVA analysis. The reasonable explanation was that SRF training may not lead a certain brain area to take an important position, which has a significant correlation change with all voxels in the whole brain. In addition, we also speculated that some true positive brain regions may not survive the multiple comparison correction when using strict threshold to decrease false positive. In our longitudinal fALFF and ReHo investigation, the values of Cerebelum_6_R (right cerebellum posterior lobe VI) and Vermis_3 (cerebellum anterior lobe III) were significantly increased with the training time. Besides, Cerebelum_6_R and Vermis_3 test values changes were significantly correlated with motor function improvement. Therefore, we speculated that a strong compensatory mechanism existed between the using SRF and changing cerebellum. When the human body adds a supernumerary sixth finger, the brain will undertake greater load and will recruit more somatomotor and sensorimotor networks to participate. Cerebellar lobules IV–VI and VIII were proven to engage in motor processing and activated in the task-based fMRI analysis (Stoodley and Schmahmann, 2009; Stoodley et al., 2010; Keren-Happuch et al., 2014), and the anterior lobe was engaged in overt limb movements (Rijntjes et al., 1999; Bushara et al., 2001). The study of the cerebellum resting-state function connectivity found that cerebellar lobules I–VI have a high correlation with the sensorimotor area (Stoodley et al., 2016; Guell et al., 2018), and then the anterior lobe III was also correlated with the somatomotor networks (Buckner et al., 2011). Therefore, the increased activation in Cerebelum_6_R and Vermis_3 was used to compensate the extra brain load caused by the supernumerary robotic finger. However, activation showed different trends in the two brain areas with the change in training time. The activation in Cerebelum_6_R increased with training time and decreased in the follow-up period (t3), which indicated that the SRF training did not cause long-lasting effects (as shown in Figure 3B). This result shows that the compensation effect of the sensorimotor area mapped in the cerebellum will return to the baseline stage with the cessation of training, but in Vermis_3, the activation increased in the early training stage (t0 to t1), decreased in the post-training stage (t1 to t2), and kept steady in the follow-up period (as shown in Figure 4B). This result shows that the somatomotor network mapped in the cerebellum plays a compensation role in the early training stage (t0 to t1). With the SRF gradually accepted, the compensation effect decreased by degrees in the post-training stage (t1 to t2), but this effect still maintained a higher level than the baseline (t0) in the post-training stage (t2) and follow-up period (t3), which proved that SRF training generated long-lasting effects.

Compared with the increased activation of the cerebellum posterior lobe VI and anterior lobe III, the decreased ReHo values in Cerebelum_9_R (right cerebellum posterior lobe IX) was also a significant result in this research (as shown in Figure 4A). Resting-state fMRI found that lobule IX has a high correlation with the default mode network, which was suppressed during tasks that demand external attention and was active during remembering, envisioning the future, and making social inferences (Buckner et al., 2011; Buckner and DiNicola, 2019). Therefore, we speculated that the decreased activation in the default mode network mapped in the cerebellum may be related with the suppression mechanism during tasks that demand external attention. The SRF as the added finger needs additional attention resources of the human brain to control, and Cerebelum_9_R could be inhibited in this active task to achieve the control purpose. As for the suppression mechanism of the default network, the reliable explanation was the significant competition effect that existed between the extensive information processing modes, which were supported by different independent network groups (Stoodley and Schmahmann, 2009). In addition, this suppression mechanism in Cerebelum_9_R was enhanced with training time and recovered in the follow-up period (t3), which indicated that the SRF training did not cause long-lasting effects (as shown in Figure 4B).

After 4 weeks of brain-controlled SRF training, this study innovatively found that the changes in the resting state of the human brain were mainly on the cerebellum. Some factors should be taken into account for this innovative result. (1) The present study takes normal people as the subject who have full use of hands in daily life. Such an experimental arrangement can better explore the neuroplasticity effect in motor augmentation of SRF training in the daily lives of normal people. This experiment arrangement may be one of the reasons for this innovative result, which is different from others. (2) The different imaginary paradigm is also one of the reasons for this innovative result. This present paradigm focused on the device of SRF and was different from the previous imaginary paradigm, which mainly focused on inborn inherent limbs (IIL) (Varkuti et al., 2013). Using IIL as the imaginary paradigm has been proven to activate the limb execution network like the somatomotor and sensorimotor network (Hardwick et al., 2018). Besides, the body part involved in the movements and the nature of the MI tasks all seem to influence the consistency of activation within the general MI network (Hetu et al., 2013). However, different from motor imagery of the IIL, using the supernumerary robot limb device, imaginary as the paradigm, was also proven to modulate other brain areas (Penaloza and Nishio, 2018). Therefore, we speculated that the new motor imagery paradigm has the potential to activate the new targeted area of the brain. (3) Unlike motor imagery training, this study mainly explored the neuroplasticity effect of the whole SRF system integrated motor imagery, extra robotic finger, and electrical stimulation feedback. This mixed influence may be different from each effect.

The difference between training with SRF and pure finger movement training on the resting-state neuroplasticity is also a very interesting question. Therefore, we preliminary arranged 2 weeks of pure finger sequence moving training experiment of nine subjects, and the training dose was matched to the SRF group. Although the behavioral performance has a significant improvement (Figure 2B), we have not found any significant changes in this control group after paired *t*-test and GRF correction in the ALFF, ReHo, and DC metrics of resting state in the cerebellum. Therefore, we inferred that the results of the SRF group were produced by the joint effect of the SRF with our body, and pure finger movement training will not cause this similar result. The results of the control group were different from the previous study in Ref. (Sale et al., 2017), and this may be related with the different duration and intensity of training or the different data process methods. In view of the preliminary implementation of this control experiment, the detailed effect needs to be further explored in the future.

Several limitations need to be noted in this study. First of all, the sample size was not large, which may limit the generalization power. More participants should be recruited to validate and extend the findings of this study. Second, it would be better to differentiate the effect caused by the motor imagery and SRF finger training. In the current study, we cannot tell the key part contributing to the neurological changes and functional recovery under the joining of the two components. In further studies, a control group is needed. Third, the effect of different task paradigms and training duration need to be further investigated. Moreover, based on the present results of behavioral performance and cerebellar neuroplasticity, the BCI-controlled SRF can be applied to the rehabilitation of patients with cerebellar stroke or functional impairment in the future.

## Conclusion

To the best of our knowledge, this is the first study to investigate brain alterations in long-term BCI-controlled supernumerary robotic finger training. Significant changes were found in Cerebelum_6_R, Cerebelum_9_R, and Vermis_3 using fALFF, ReHo, and DC metrics in longitudinal resting-state fMRI study. In addition, fALFF value changes in Cerebelum_6_R and ReHo value changes in Vermis_3 were significantly correlated with motor function improvement. We conclude that the compensation mechanism of the sensorimotor and somatomotor networks mapped in the cerebellum existed during BCI-controlled SRF training. At the same time, the suppression mechanism was also observed in the default mode network mapped in the cerebellum in this study. Our new findings supplement the literature on motor-augmentation neuroplasticity brought by BCI-controlled augmentative device training and may facilitate future research on SRF.

## Data Availability Statement

The raw data supporting the conclusions of this article will be made available by the authors, without undue reservation.

## Ethics Statement

The studies involving human participants were reviewed and approved by the Tianjin University Human Research Ethics Committee. The patients/participants provided their written informed consent to participate in this study. Written informed consent was obtained from the individual(s) for the publication of any potentially identifiable images or data included in this article.

## Author Contributions

YL originally conceptualized the study and drafted the manuscript. SH designed the experiment and analyzed the fMRI data. ZW provided the EEG coding and decoding of the algorithms. FJ designed the hardware system of the SRF. DM gave some advice on hand prehensile taxonomy and helped review the content of the manuscript. All authors contributed to the article and approved the submitted version.

## Conflict of Interest

The authors declare that the research was conducted in the absence of any commercial or financial relationships that could be construed as a potential conflict of interest.

## Publisher’s Note

All claims expressed in this article are solely those of the authors and do not necessarily represent those of their affiliated organizations, or those of the publisher, the editors and the reviewers. Any product that may be evaluated in this article, or claim that may be made by its manufacturer, is not guaranteed or endorsed by the publisher.
